# A Case of Capsular Secondary Cataract with Calcareous Deposit on Anterior Capsule, Following Operation by Division

**Published:** 1869-07

**Authors:** Eugene Smith

**Affiliations:** Detroit


ART. III.—*A Case of Capsular Secondary Cataract with Calcareous Deposit on Anterior Capsule, following Operation by Division.* BY EUGENE SMITH, M. D., *Detroit.*

Capsular secondary cataract after operations by extraction or division, is very common and frequently met with by all ophthalmic surgeons, who are also aware of the occasional presence of lime in dust-like molecules, which unite and form large grains, and quite often, especially where the cataract originates from inflammation, occurs in large concrements in the cataractous mass or lens substance, but the formation of calcareous matter, and subsequently of capsular cataract must be very rare, as it is not spoken of by our best authors on ophthalmology, and has never been observed by either of several oculists of large experience, with whom I have spoken on the subject. The particulars of the case are as follows:

Miss C., aged 29 years, of a nervous temperament, consulted me with regard to an operation for cataract in both eyes; she, as her parents say, having been born so. An examination of the eyes disclosed simply soft, total cataract of each eye, with no appearance of chalky matter in either. As there was no evidence of retinal disease, I advised an operation. The operation, which was made in May, 1868, was by division, and was very successful, there being at the end of a month good vision, and not a vestige of the lens to be seen, and no indications of capsular opacity. I pronounced her well, and advised glasses, with which she saw nicely. For a month or six weeks I did not see the case, when she called and said the cataract was "coming back," in the right eye. On examination I discovered several white points, evidently forming on the capsule, which was perfectly transparent. I dilated the pupil and by oblique illumination could see quite a number of points, which appeared to be on the anterior capsule, and below the axis of vision. As they did not interfere much with sight I did nothing, but watched the case, seeing it every few days, for seven or eight months, during which time the deposit increased in size and the capsule became opaque, necessitating an operation for its removal, which was done by extraction in May, 1869, one year from the date of first operation. During the time of its first formation there were no symptoms of inflammation. It is now one month since the last operation, and the eyes seem as perfect as any, after being operated upon for cataract, aside from a considerable degree of nystagmus, which is improving. The patient has commenced going to school, and I hear is learning to read.

The deposit is no doubt of a calcareous nature; it is hard, gritty, and of a whitish-yellow color. In size it is about one-half a line in thickness, about two lines in length in its longest, and one line in its shortest diameter.

